# Supplementary material for: The Small RNA Universe of Capitella teleta
Source: Front Mol Biosci. 2022 Feb 25;9:802814. doi: 10.3389/fmolb.2022.802814 (PMC8915122; doi:10.3389/fmolb.2022.802814)
Supplement: Supplementary file 1 [file DataSheet1.ZIP › Supplement/known/CAPTEscaffold_127_10582.pdf]

Provisional ID	:	CAPTEscaffold_127_10582
Score total	:	5.2
Score for star read(s)	:	-1.3
Score for read counts	:	0
Score for mfe	:	1.9
Score for randfold	:	1.6
Score for cons. seed	:	3
Total read count	:	304624
Mature read count	:	292694
Loop read count	:	0
Star read count	:	11930

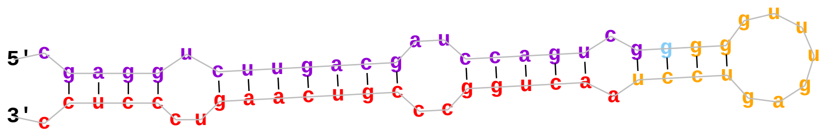

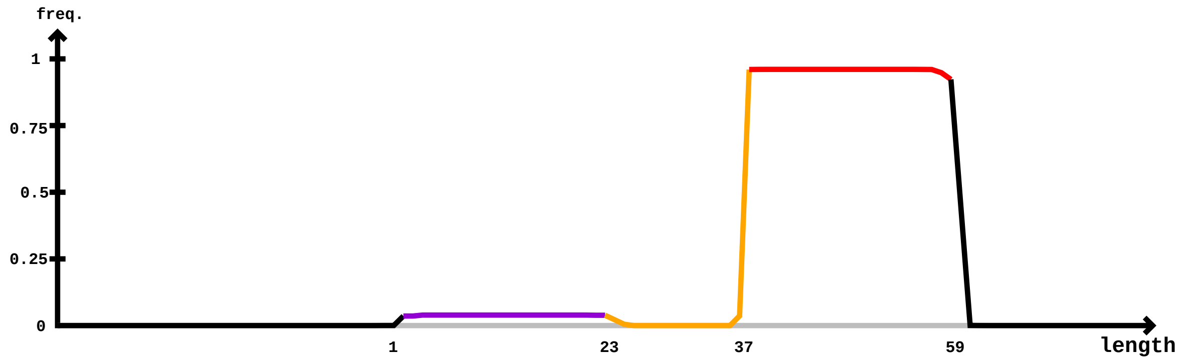


```
                                           Star                                   Mature
5'- aucauccgacauggacacuugacguggcauaagacgaggucuugacgauccagucggggguuugaguccuaacuggcccgucaagucccucccaaugccacggccuacuuca   -3'   obs
    aucauccgacauggacacuugacguggcauaagacgaggucuugacgauccagucggggguuugaguccuaacuggcccgucaagucccucccaaugccacggccuacuuca         exp
    aucauccgacauggacacuugacguggcauaagacgaggucuugacgauccagucggggguuugaguccuaacuggcccgucaagucccucccaaugccacggccuacuuca         known
    ....(((.....))).......((((((((.....((((.(((((((..(((((.((((.......)))).)))))..)))))))..))))...))))))))..........   reads   mm   sample
    ................................gacgaggucuugacgauccag...........................................................   5       0    seq
    ................................gacgaggucuugacgauccagu..........................................................   46      0    seq
    ................................gNcgaggucuugacgauccagu..........................................................   1       1    seq
    ................................gacgaggucuCgacgauccagu..........................................................   1       1    seq
    .................................acgaggucuugacgauccagu..........................................................   1       0    seq
    ..................................cgaggucuugacgauccag...........................................................   2       0    seq
    ..................................cgaggucAugacgauccagu..........................................................   1       1    seq
    ..................................cgaggucuugacgauccagu..........................................................   129     0    seq
    ..................................cgaggucuugacgaAccagu..........................................................   1       1    seq
    ..................................cgaggucuugacgauccaguc.........................................................   25      0    seq
    ..................................cgaggucuugacAauccagucg........................................................   11      1    seq
    ..................................cgaggucuugacgauccUgucg........................................................   1       1    seq
    ..................................cgaggucuugacgauccaguAg........................................................   1       1    seq
    ..................................cgaggucuugacgauccagGcg........................................................   1       1    seq
    ..................................cgaggucuugacgaucUagucg........................................................   6       1    seq
    ..................................cgaggucuugacgauccagAcg........................................................   1       1    seq
    ..................................cgagCucuugacgauccagucg........................................................   1       1    seq
    ..................................cgaggucuugGcgauccagucg........................................................   2       1    seq
    ..................................cgagAucuugacgauccagucg........................................................   3       1    seq
    ..................................Ugaggucuugacgauccagucg........................................................   4       1    seq
    ..................................cCaggucuugacgauccagucg........................................................   1       1    seq
    ..................................cgaggucuugacgGuccagucg........................................................   2       1    seq
    ..................................cgaggucuugacgauccagucA........................................................   3       1    seq
    ..................................cUaggucuugacgauccagucg........................................................   1       1    seq
    ..................................cgaggucuugacgaucAagucg........................................................   3       1    seq
    ..................................cgaggucuugacgaAccagucg........................................................   2       1    seq
    ..................................cgaggucuugacgaCccagucg........................................................   2       1    seq
    ..................................cgaAgucuugacgauccagucg........................................................   5       1    seq
    ..................................cgagguUuugacgauccagucg........................................................   1       1    seq
    ..................................cgaggucuugacgauccagucg........................................................   5267    0    seq
    ..................................cgaggucuugacgCuccagucg........................................................   1       1    seq
    ..................................cgaggucuugacgauAcagucg........................................................   2       1    seq
```

```
                                  Star                              Mature
aucauccgacauggacacuugacguggcauaagacgaggucuugacgauccagucgggguuugaguccuaacuggcccgucaagucccucccaaugccacggccuacuuca
..................................cgaggucAugacgauccagucg........................................................  2     1   seq
..................................cgaggucuugaAgauccagucg........................................................  1     1   seq
..................................cgaggucuugacgauccagucC........................................................  8     1   seq
..................................cgaggucuugacgauccagucU........................................................  61    1   seq
..................................cgaggucuAgacgauccagucg........................................................  4     1   seq
..................................cgaggucuugacgauccagCcg........................................................  1     1   seq
..................................cAaggucuugacgauccagucg........................................................  20    1   seq
..................................cgagguAuugacgauccagucg........................................................  2     1   seq
..................................Agaggucuugacgauccagucg........................................................  1     1   seq
..................................cgaggAcuugacgauccagucg........................................................  1     1   seq
..................................cgaggucuuCacgauccagucg........................................................  2     1   seq
..................................cgaggucuugacgaGccagucg........................................................  1     1   seq
..................................cgaggucuCgacgauccagucg........................................................  2     1   seq
..................................cgaggucuugacgauccaguUg........................................................  3     1   seq
..................................cgaggucuugacgauccagucgg.......................................................  3795  0   seq
..................................cgaggucGugacgauccagucgg.......................................................  1     1   seq
..................................cgaggucuugacgauccagucgA.......................................................  18    1   seq
..................................cgaggucuugacgauUcagucgg.......................................................  1     1   seq
..................................cgaggucuAgacgauccagucgg.......................................................  1     1   seq
..................................cgaggucuugGcgauccagucgg.......................................................  1     1   seq
..................................cgGggucuugacgauccagucgg.......................................................  1     1   seq
..................................cgaggucuugacgauccagGcgg.......................................................  1     1   seq
..................................cgaggucuugaUgauccagucgg.......................................................  2     1   seq
..................................cgaggucuugacgaucUagucgg.......................................................  5     1   seq
..................................cAaggucuugacgauccagucgg.......................................................  2     1   seq
..................................Agaggucuugacgauccagucgg.......................................................  1     1   seq
..................................cgaggucuugacgaucAagucgg.......................................................  1     1   seq
..................................cgaggucuugacgauAcagucgg.......................................................  1     1   seq
..................................cgaggucuugacgauccaguUgg.......................................................  1     1   seq
..................................cgaggucuugacgauccaAucgg.......................................................  1     1   seq
..................................cgaggucuugacgaucGagucgg.......................................................  1     1   seq
..................................cgaggucuCgacgauccagucgg.......................................................  2     1   seq
..................................cgaggucuugacgauccagucgC.......................................................  1     1   seq
..................................cgaggucuugacgauccaguAgg.......................................................  1     1   seq
..................................cgUggucuugacgauccagucgg.......................................................  1     1   seq
..................................cgaggucuugacgauccagucgU.......................................................  367   1   seq
..................................cgaggucuugacAauccagucgg.......................................................  9     1   seq
..................................cgaggucuugacgaucUagucggg......................................................  1     1   seq
..................................cgaggucuugGcgauccagucggg......................................................  1     1   seq
..................................cgaggucuugacgauccaguGggg......................................................  1     1   seq
..................................cgaggucuugacgauccagucggg......................................................  152   0   seq
..................................cgaggucuugacgauccCgucggg......................................................  1     1   seq
..................................cgaggucuugaAgauccagucggg......................................................  1     1   seq
..................................cgaggucuugacgauccagucggA......................................................  249   1   seq
..................................cgaggucuugacgauccagucggU......................................................  594   1   seq
..................................cgaggucuugacgauccagucgggA.....................................................  14    1   seq
..................................cgaggucuugacgauccagucgggU.....................................................  17    1   seq
...................................gaggucuugacgauccagucg........................................................  17    0   seq
...................................gaggucuugacgauccagucUg.......................................................  1     1   seq
...................................Aaggucuugacgauccagucgg.......................................................  1     1   seq
...................................gaggucuugacgauccagucgg.......................................................  4     0   seq
...................................gaggucuugacgauccagucgU.......................................................  1     1   seq
...................................gaggucuugacgauccagucggA......................................................  1     1   seq
....................................aggucuugacgauccagu..........................................................  12    0   seq
....................................aggucuugacgauccaguc.........................................................  4     0   seq
....................................aggAcuugacgauccagucg........................................................  1     1   seq
....................................aggucuugacgauccagucg........................................................  63    0   seq
....................................aggucuugacAauccagucgg.......................................................  1     1   seq
....................................aggucuugacgauccagucgU.......................................................  98    1   seq
....................................Nggucuugacgauccagucgg.......................................................  1     1   seq
....................................aggGcuugacgauccagucgg.......................................................  1     1   seq
....................................aCgucuugacgauccagucgg.......................................................  1     1   seq
....................................aggucuugacgauccagucgg.......................................................  453   0   seq
....................................aggucuugacgaucUagucgg.......................................................  1     1   seq
....................................aggucuugacgauccagucggU......................................................  169   1   seq
....................................aggucuugacgauccagucggA......................................................  262   1   seq
....................................aggucuugacgauccagucgggU.....................................................  1     1   seq
.....................................ggucuugacgauccagucg........................................................  3     0   seq
....................................................................cuaacuggcccgucaagucccucc....................  3     0   seq
.....................................................................uaacuggcccgucaaguc.........................  1     0   seq
```

```
                                   Star                                Mature
aucauccgacauggacacuugacguggcauaagacgaggucuugacgauccagucgggggguuugaguccuaacuggcccgucaagucccucccaaugccacggccuacuuca
......................................................................uaacuggcccgucaagucc........................   16     0   seq
......................................................................uaacuggcccgucaaguccc.......................   9      0   seq
......................................................................uaacuggccUgucaagucccu......................   2      1   seq
......................................................................uaacuggcAcgucaagucccu......................   1      1   seq
......................................................................Naacuggcccgucaagucccu......................   1      1   seq
......................................................................uaacuggcccguAaagucccu......................   1      1   seq
......................................................................uaacAggcccgucaagucccu......................   1      1   seq
......................................................................uaacuggcccgucaagucccu......................   417    0   seq
......................................................................uaacuggcccgAcaagucccu......................   1      1   seq
......................................................................uaacuggcccgucaaguccc G......................   3      1   seq
......................................................................uaacuggcccgucaagucccC......................   2      1   seq
......................................................................Aaacuggcccgucaagucccu......................   2      1   seq
......................................................................uaacuggcccgucaagucUcuc.....................   1      1   seq
......................................................................Gaacuggcccgucaagucccuc.....................   1      1   seq
......................................................................uaacuggcccgucaagucccuc.....................   358    0   seq
......................................................................uaacuggGccgucaagucccuc.....................   1      1   seq
......................................................................uaacuggcccgucGagucccuc.....................   1      1   seq
......................................................................uaacuggcccgucaagucccuU.....................   18     1   seq
......................................................................Aaacuggcccgucaagucccuc.....................   3      1   seq
......................................................................uaacuggcccgucaagAcccuc.....................   1      1   seq
......................................................................uaUcuggcccgucaagucccucc....................   1      1   seq
......................................................................uaacuggcccCucaagucccucc....................   1      1   seq
......................................................................uaacuggcccgucGagucccucc....................   2      1   seq
......................................................................uaacuggcccgucaUgucccucc....................   1      1   seq
......................................................................uaacuggcccgucaaguAccucc....................   1      1   seq
......................................................................uaacuggcccgucaagucccuGc....................   1      1   seq
......................................................................uaacuggcccgucaagucccucc....................   8836   0   seq
......................................................................uaacuggcccgucaagucAcucc....................   9      1   seq
......................................................................uaacuggcccgucaagucccucA....................   7      1   seq
......................................................................uaacuggcccAucaagucccucc....................   2      1   seq
......................................................................uaacuggcccgucaagucccucU....................   35     1   seq
......................................................................uaacuggcccgGcaagucccucc....................   1      1   seq
......................................................................uaacuggcccgucaagucccuAc....................   3      1   seq
......................................................................uaacuggcccgucaagucccCcc....................   1      1   seq
......................................................................uaacuggcccgucCagucccucc....................   1      1   seq
......................................................................uaacuggcccgucaagucccAcc....................   2      1   seq
......................................................................uaacuggcccgCcaagucccucc....................   4      1   seq
......................................................................uaacuggcccgucaagAcccucc....................   3      1   seq
......................................................................Naacuggcccgucaagucccucc....................   3      1   seq
......................................................................uaacuggcccgucaaguUccucc....................   4      1   seq
......................................................................uaacuggcccgucaaguccAucc....................   2      1   seq
......................................................................uaacuggccUgucaagucccucc....................   9      1   seq
......................................................................Caacuggcccgucaagucccucc....................   93     1   seq
......................................................................uaacuggcccgucaaCucccucc....................   1      1   seq
......................................................................uaacuggccGgucaagucccucc....................   1      1   seq
......................................................................uaacuggGccgucaagucccucc....................   1      1   seq
......................................................................uaacuggcccgucaaAucccucc....................   1      1   seq
......................................................................uaacuggcccguUaagucccucc....................   1      1   seq
......................................................................uaacuggcccUucaagucccucc....................   2      1   seq
......................................................................uaacuggcAcgucaagucccucc....................   3      1   seq
......................................................................uaacuAgcccgucaagucccucc....................   5      1   seq
......................................................................uaacuggcccgucaaguGccucc....................   2      1   seq
......................................................................uaacuggcccgucaaguccUucc....................   5      1   seq
......................................................................Aaacuggcccgucaagucccucc....................   54     1   seq
......................................................................uaacuggcccgucaagucUcucc....................   8      1   seq
......................................................................uaacAggcccgucaagucccucc....................   14     1   seq
......................................................................uaacuUgcccgucaagucccucc....................   1      1   seq
......................................................................Gaacuggcccgucaagucccucc....................   3      1   seq
......................................................................uaacuggcUcgucaagucccucc....................   3      1   seq
......................................................................uGacuggcccgucaagucccucc....................   1      1   seq
......................................................................uaacuggcccguAaagucccucc....................   3      1   seq
......................................................................uaaUuggcccgucaagucccucc....................   1      1   seq
......................................................................uaacuggccAgucaagucccucc....................   6      1   seq
......................................................................uaacuggcccgucaagucccGcc....................   1      1   seq
......................................................................uaacugUcccgucaagucccucc....................   3      1   seq
......................................................................uaacuggcccgAcaagucccucc....................   2      1   seq
......................................................................uaacuggcccgucaagucccuccA...................   5      1   seq
......................................................................uaacuggcccgucaagucccuccU...................   1096   1   seq
......................................................................uaacuggcccgucaagucccuccG...................   1      1   seq
......................................................................uaacuggcccgucaagucccuccAa..................   3      1   seq
```

Star Mature

```
aucauccgacauggacacuugacguggcauaagacgaggucuugacgauccagucggggguuugaguccuaacuggcccgucaagucccucccaaugccacggccuacuuca
.....................................................................uaacuggcccgucaagucccuccAaa.................    1     1    seq
......................................................................aacuggcccgucaagucc........................    34    0    seq
......................................................................aacuggcccgucaaguccc.......................    73    0    seq
......................................................................Gacuggcccgucaaguccc.......................    1     1    seq
......................................................................aacuggcccgucaagucUc.......................    1     1    seq
......................................................................aacuggccGgucaagucccu......................    1     1    seq
......................................................................aacuggcccgucaGgucccu......................    1     1    seq
......................................................................aacuggAccgucaagucccu......................    5     1    seq
......................................................................aacuggcccAucaagucccu......................    2     1    seq
......................................................................aacuggcccgucaagucccA......................    2     1    seq
......................................................................aacuggcUcgucaagucccu......................    2     1    seq
......................................................................aUcuggcccgucaagucccu......................    2     1    seq
......................................................................aacuggccUgucaagucccu......................    3     1    seq
......................................................................aacuggcccgucaagucccG......................    15    1    seq
......................................................................aacCggcccgucaagucccu......................    1     1    seq
......................................................................Nacuggcccgucaagucccu......................    2     1    seq
......................................................................aacuggcccgucaagCcccu......................    31    1    seq
......................................................................aacuggcccguUaagucccu......................    1     1    seq
......................................................................aacuggcccguAaagucccu......................    1     1    seq
......................................................................aacuggcccgucaagucccu......................    3070  0    seq
......................................................................aGcuggcccgucaagucccu......................    1     1    seq
......................................................................aacuggcccgucGagucccu......................    1     1    seq
......................................................................aacuggcccgucaaguAccu......................    1     1    seq
......................................................................aacuggcccgAcaagucccu......................    1     1    seq
......................................................................aacuggcccgucaagucGcu......................    1     1    seq
......................................................................Gacuggcccgucaagucccu......................    4     1    seq
......................................................................aacuggcccgucaagucccC......................    27    1    seq
......................................................................aaUuggcccgucaagucccu......................    3     1    seq
......................................................................aacuggcccUucaagucccu......................    1     1    seq
......................................................................aacuggcccgucaaguccUu......................    2     1    seq
......................................................................aacuggcccgucaagucUcu......................    1     1    seq
......................................................................aacAggcccgucaagucccu......................    1     1    seq
......................................................................aacuggcAcgucaagucccu......................    2     1    seq
......................................................................aacuggcccgucaaguUccu......................    1     1    seq
......................................................................aacuggcccgucaagucccuA.....................    2     1    seq
......................................................................aacugUcccgucaagucccuc.....................    1     1    seq
......................................................................aNcuggcccgucaagucccuc.....................    2     1    seq
......................................................................aacuggGccgucaagucccuc.....................    1     1    seq
......................................................................aacuggcccguAaagucccuc.....................    1     1    seq
......................................................................aacuggcccgucaaguAccuc.....................    2     1    seq
......................................................................aacuggcccgucGagucccuc.....................    1     1    seq
......................................................................aacuggcccgucaagucccCc.....................    9     1    seq
......................................................................aacuggcccgucaagucAcuc.....................    6     1    seq
......................................................................aacuggcccAucaagucccuc.....................    2     1    seq
......................................................................aacuggcccgucaagucccuU.....................    109   1    seq
......................................................................aacugAcccgucaagucccuc.....................    2     1    seq
......................................................................aacuggcccgucaaguUccuc.....................    1     1    seq
......................................................................aacuggccGgucaagucccuc.....................    1     1    seq
......................................................................aacuggcccgAcaagucccuc.....................    2     1    seq
......................................................................aacuggcccgucaagucccGc.....................    1     1    seq
......................................................................aacuggcccgucaagucccuG.....................    1     1    seq
......................................................................aacuggcccgucUagucccuc.....................    1     1    seq
......................................................................aacuggcccgucaagucccAc.....................    1     1    seq
......................................................................aacuggcccgucaagAcccuc.....................    6     1    seq
......................................................................aGcuggcccgucaagucccuc.....................    1     1    seq
......................................................................aacuggcccCucaagucccuc.....................    1     1    seq
......................................................................aacuggcccgucaagucUcuc.....................    6     1    seq
......................................................................aacuggcccgucaGgucccuc.....................    2     1    seq
......................................................................aacuggUccgucaagucccuc.....................    3     1    seq
......................................................................aacuggcAcgucaagucccuc.....................    2     1    seq
......................................................................aacuggcccgGcaagucccuc.....................    2     1    seq
......................................................................aaAuggcccgucaagucccuc.....................    1     1    seq
......................................................................Nacuggcccgucaagucccuc.....................    1     1    seq
......................................................................Gacuggcccgucaagucccuc.....................    5     1    seq
......................................................................aacuAgcccgucaagucccuc.....................    4     1    seq
......................................................................aacuggcccgucaagucccuc.....................    7054  0    seq
......................................................................aacuggcccgCcaagucccuc.....................    1     1    seq
......................................................................aacuggcccgucaaAucccuc.....................    1     1    seq
......................................................................aacuggAccgucaagucccuc.....................    12    1    seq
......................................................................aacuggcGcgucaagucccuc.....................    1     1    seq
```

Star **Mature**

aucauccgacauggacacuugacguggcauaagacgaggucuugacgauccagucggggguuugaguccuaacuggcccgucaagucccucccaaugccacggccuacuuca			
....aacuggcccguUaagucccuc.....................	1	1	seq
....aacuggccUgucaagucccuc.....................	10	1	seq
....aacuggcUcgucaagucccuc.....................	3	1	seq
....aacCggcccgucaagucccuc.....................	2	1	seq
....aacAggcccgucaagucccuc.....................	4	1	seq
....aacuggcccgucaagCcccuc.....................	2	1	seq
....aacuggcccgucaaguccUuc.....................	5	1	seq
....aacuggcccgucaaAucccucc....................	24	1	seq
....aacuggcccgucaaguGccucc....................	23	1	seq
....aNcuggcccgucaagucccucc....................	9	1	seq
....aacuggcccgCcaagucccucc....................	39	1	seq
....aacuggcccgucaagucUcucc....................	173	1	seq
....aaGuggcccgucaagucccucc....................	5	1	seq
....aacuggccGgucaagucccucc....................	14	1	seq
....aacuggcccgucaagGcccucc....................	4	1	seq
....aacuAgcccgucaagucccucc....................	153	1	seq
....aGcuggcccgucaagucccucc....................	33	1	seq
....aacuggcccguAaagucccucc....................	63	1	seq
....aaUuggcccgucaagucccucc....................	12	1	seq
....aacCggcccgucaagucccucc....................	29	1	seq
....aacuggcccgucaaguccAucc....................	40	1	seq
....aacuggcccAucaagucccucc....................	71	1	seq
....aacAggcccgucaagucccucc....................	175	1	seq
....aacuggcccgucaaUucccucc....................	8	1	seq
....aacuggcccgucUagucccucc....................	22	1	seq
....Cacuggcccgucaagucccucc....................	9	1	seq
....aacuggcccgucaagucccucc....................	233118	0	seq
....aacuggcccgucaaCucccucc....................	15	1	seq
....aacuggcccgucaagucccucA....................	176	1	seq
....aacuggccUgucaagucccucc....................	213	1	seq
....aacuggcccgucaagAcccucc....................	112	1	seq
....Gacuggcccgucaagucccucc....................	169	1	seq
....aacuggcccgucaagucccuGc....................	9	1	seq
....aCcuggcccgucaagucccucc....................	1	1	seq
....aacuggcccgucaUgucccucc....................	18	1	seq
....aacuggcccgAcaagucccucc....................	113	1	seq
....aacuggcccgucaagucccuUc....................	62	1	seq
....aacuggcUcgucaagucccucc....................	76	1	seq
....Nacuggcccgucaagucccucc....................	75	1	seq
....aacuggcccgucaagucGcucc....................	22	1	seq
....aacuggcGcgucaagucccucc....................	17	1	seq
....aacuUgcccgucaagucccucc....................	5	1	seq
....aacuggAccgucaagucccucc....................	140	1	seq
....aacuggGccgucaagucccucc....................	37	1	seq
....aacuggcccgucaCgucccucc....................	2	1	seq
....aacuggcAcgucaagucccucc....................	62	1	seq
....aacuggcccgucaagucccuAc....................	89	1	seq
....aacuggcccgucaaguUccucc....................	59	1	seq
....aacuggcccgucaagucccucG....................	15	1	seq
....aaAuggcccgucaagucccucc....................	54	1	seq
....aacuggcccgucaagucccGcc....................	10	1	seq
....aacuggcccNucaagucccucc....................	2	1	seq
....aacuggccAgucaagucccucc....................	59	1	seq
....aacuggcccgucaagucAcucc....................	168	1	seq
....aacuggcccgucaagucccCcc....................	26	1	seq
....aacuggcccgGcaagucccucc....................	12	1	seq
....aacuggcccgucaagucccAcc....................	48	1	seq
....aacuCgcccgucaagucccucc....................	28	1	seq
....aacuggcccUucaagucccucc....................	10	1	seq
....aacuggcccgucCagucccucc....................	5	1	seq
....aacuggcccguGaagucccucc....................	11	1	seq
....aacuggcccgucaGgucccucc....................	27	1	seq
....aacugAccgucaagucccucc....................	52	1	seq
....Uacuggcccgucaagucccucc....................	18	1	seq
....aacuggUccgucaagucccucc....................	68	1	seq
....aUcuggcccgucaagucccucc....................	17	1	seq
....aacugCcccgucaagucccucc....................	28	1	seq
....aacuggcccCucaagucccucc....................	10	1	seq
....aacGggcccgucaagucccucc....................	4	1	seq
....aacuggcccgucaaguccGucc....................	10	1	seq

Star Mature

```
aucauccgacauggacacuugacguggcauaagacgaggucuugacgauccagucggggguuugaguccuaacuggcccgucaagucccucccaaugccacggccuacuuca
......................................................................aacugUcccgucaagucccucc....................  21     1   seq
......................................................................aacuggcccgucaagucccucU....................  2497   1   seq
......................................................................aacugNcccgucaagucccucc....................  2      1   seq
......................................................................aacuggcccguUaagucccucc....................  64     1   seq
......................................................................aacuggcccgucaaguccUucc....................  99     1   seq
......................................................................aacuggcccgucaaguAccucc....................  83     1   seq
......................................................................aacuggcccgucaagCcccucc....................  48     1   seq
......................................................................aacuggcccgucGagucccucc....................  54     1   seq
......................................................................aacuggcccgucaagucccuccG...................  30     1   seq
......................................................................aacuggcccgucaagucccucUc...................  1      1   seq
......................................................................aacuggcccgucaagucccuccA...................  328    1   seq
......................................................................aacuggcccgucaagucccuccc...................  32     0   seq
......................................................................aacuggcccgucaagucccuccU...................  31364  1   seq
......................................................................aacuggcccgucaagucccucccU..................  7      1   seq
......................................................................aacuggcccgucaagucccuccUa..................  19     1   seq
......................................................................aacuggcccgucaagucccuccAa..................  49     1   seq
......................................................................aacuggcccgucaagucccuccUaa.................  2      1   seq
......................................................................aacuggcccgucaagucccuccAaa.................  21     1   seq
......................................................................aacuggcccgucaagucccucccaa.................  2      0   seq
.......................................................................Ccuggcccgucaagucccu......................  1      1   seq
.......................................................................acuggcccgucaagucccu......................  24     0   seq
.......................................................................acuggcccgucaagucccucc....................  16     0   seq
.......................................................................acuggcccgucaagucccucU....................  24     1   seq
.......................................................................acuggccUgucaagucccucc....................  1      1   seq
.......................................................................acuggcccgucaagucUcucc....................  1      1   seq
.......................................................................acuggcccgucaagucccuccU...................  44     1   seq
.......................................................................acuggcccgucaagucccuccA...................  3      1   seq
........................................................................cuggcccgucaagucccucc....................  6      0   seq
........................................................................cuggcccgucaagucccuccU...................  2      1   seq
........................................................................cuggcccgucaagucccuccAa..................  1      1   seq
.........................................................................uggcccgucaagucccucc....................  1      0   seq
.........................................................................uggcccgucaagucccucccU..................  2      1   seq
..........................................................................ggcccgucaagucccucc....................  2      0   seq
```
